# Pruritus Vulvæ Treated with Suphurous Acid

**Published:** 1878-11-20

**Authors:** Edward B. Stevens

**Affiliations:** Lebanon, Ohio


### PRURITUS VULVÆ TREATED WITH SUPHUROUS ACID.

BY EDWARD B. STEVENS, M. D. LEBANON, OHIO.

I was recently consulted by a lady complaining as follows: Severe pruritus of the labial surfaces, extending to the external genitals, with an erysipelatous rash covering these surfaces, and at the same time an abundant leucorrhœal discharge. She had applied a variety of lotions to the itching, burning parts without avail:—the leucorrhaœ had been of some time standing; general health good; supposes herself approaching the menopause, age 46.

Upon examination found an erysipelatous rash covering the labia and flaming up over the pubic region towards, the lower surface of the abdomen; it was angry looking and eczematous, with a watery exudation; on introducing the speculum found the rash occupying the labial surfaces and extending up over the outlet of the vagina. The superior portion of the vagina, and cervix of the uterus were perfectly healthy in appearance, whereas I had expected to find abrasion of the os, or some condition of chronic inflamation as the reason for the leucorrhœ discharge. Instead, I found the red point of a small mucous polypus about the size of a large pea showing itself at the os. I had no difficulty in grasping the pedicle of this small polypus with slender forceps and snipping it off with scissors. I supposed this polypus was the irritant that produced the leucorrhœa—and as I expected, its removal almost entirely arrested the discharge.

For the pruitus and burning, I directed the parts to be freely bathed with sulphurous acid in full strength. The result was a prompt and entire relief. Subsequently there was a partial return for several times of the rash and puritus, but always completely and promptly relieved as at first by the free application of the sulphurous acid.

My attentien was called to the efficacy of sulphurous acid in kindred cutaneous troubles, by a paper read a year ago in the American Dermatological Association by Dr. L. D. Bulkley of New York. He regards the group of cases he describes in that paper, as not only eczematous, but as having a parasitic origin, which he found to be uniformly corrected by the application of this acid. Shortly before the present case came into my care, a lady applied to me with eczema of the face and neck, that came under the care of one of my most intelligent medical friends, and resisted all reasonable treatment, constitutional and local for many months. Dr. Bulkey's cases being fresh in my mind, I laid aside all constitutional remedies, and directed the parts to be freely bathed with sulphurous acid, full strength, with the effect to afford perfect and, as Bulkley expresses it—"exquisit relief." The acid was reapplied from time to time as the itching recurred and the cure is now complete, the skin having lost its scaly condition and become as smooth as an infant's.

Some writers direct the application of sulphurous acid variously diluted—as with water, or glycerine. My experience, in a few cases only, agrees with that of Dr. Bulkley, that there is no necessity to dilute the acid even for very delicate surfaces, I therefore direct the acid to be kept closely stopped, in bulk—and the patient to have an ounce ground glass stopper vial, which is kept supplied from the larger bottle for use ; due care being observed to avoid as far as possible atmospheric influence upon the acid. I advise the parts effected to be well saturated when ever the itching calls attention to the disease.

Puritus vulvæ is frequently an obstinate affection, but I have hitherto found cases which were evidently eczematous, and my experience in the foregoing case, is given simply as affording an additional rational therapeutic remedy, especially when the pruritus is associated with this condition of parts.—*Ex.*

